# Dose or Timing? The Role of Prophylaxis in Preventing Joint Bleeds in Hemophilia: A Cross-Generational Comparative Analysis

**DOI:** 10.5152/eurasianjmed.2026.251208

**Published:** 2026-07-16

**Authors:** Zühal Demirci, Nur Soyer, Fatma Keklik Karadağ, Ayşenur Arslan, Güray Saydam, Fahri Şahin

**Affiliations:** 1 Department of Internal Medicine , Ege University Hospital Faculty of Medicine, İzmir, Türkiye; 2 Department of Hematology, Ege University Faculty of Medicine, İzmir, Türkiye; 3 Institute of Transfusion Medicine, Ulm University, Ulm, Germany

**Keywords:** Hemophilia, hemophilic arthropathy, joint health, prophylaxis

## Abstract

**Background:**

This study aimed to assess intergenerational differences in joint health and functional independence among adults with hemophilia and to examine the relationship between prophylaxis timing, dose intensity, and treatment adherence and long-term joint outcomes.

**Methods:**

A retrospective study was conducted including 91 adult patients with hemophilia A or B. Participants were classified into generational cohorts within different treatment eras. Joint health and functional status were assessed using the Hemophilia Joint Health Score (HJHS) and the Functional Independence Score in Hemophilia (FISH). Statistical analyses included correlation analyses, subgroup comparisons, and general linear models; subgroup analyses were considered exploratory.

**Results:**

The median age was 38 years, and prophylaxis was initiated at a median age of 27 years. Patients had a mean annual bleeding rate of 11.1, with a median of 2 joints affected per year, most commonly the ankles, knees, and elbows. The median prophylaxis dose was 20.8 IU/kg, and bleeding episodes significantly decreased in patients requiring dose escalation (*P* = .007). Higher joint scores were associated with poorer functional outcomes and a greater number of bleeding joints. Joint health and functional outcomes differed significantly across generations (*P* < .001), with worse outcomes in older cohorts. Follow-up showed no significant improvement over 1 year, although baseline and follow-up joint scores were strongly correlated (*P* < .001).

**Conclusion:**

In adults with hemophilia, delayed prophylaxis initiation was associated with poorer joint and functional outcomes. The observed intergenerational differences likely reflect historical variations in access to prophylaxis and treatment practices. Although individualized dose escalation improved bleeding control, established joint damage showed limited short-term reversibility.

**Main Points**

- Early and consistent prophylaxis appeared to be more strongly associated with joint preservation and functional independence in hemophilia in this real-world cohort.
- Prophylaxis dose escalation significantly reduced bleeding frequency, indicating a potential benefit of individualized dose optimization.
- Consistent prophylaxis and better treatment adherence were associated with improved joint outcomes across all age groups.

## Introduction

The primary goal of hemophilia treatment is to prevent bleeding, joint damage, and related complications, thereby reducing treatment burden and improving quality of life. Treatment is individualized according to disease severity and bleeding status, with factor replacement therapy administered either prophylactically to prevent bleeding or on demand to treat bleeding episodes.^1^^,^^2^

To effectively prevent joint damage, prophylactic treatment should be initiated before the onset of joint disease, ideally before the first joint bleed. Long-term prophylaxis, representing a paradigm shift in the management of severe hemophilia, is highly effective in preventing hemophilic arthropathy when started at an early age.^1^ ^,^^3^^,4^ Prophylaxis is administered using different dose intensities and infusion frequencies according to established guidelines. High-, intermediate-, and low-dose prophylaxis regimens are defined based on the amount and frequency of factor VIII or IX administered and may be adjusted to prevent bleeding.^5^ The World Federation of Hemophilia (WFH) recommends early initiation of high- or intermediate-dose prophylaxis as first-line treatment for severe hemophilia because it substantially reduces bleeding compared with on-demand therapy. In resource-limited settings, low-dose prophylaxis has been proposed as a feasible alternative and has been associated with reduced bleeding and improved joint outcomes compared with episodic treatment. Historical differences in treatment intensity and access may therefore contribute to variability in long-term joint outcomes across treatment eras.^6^ Cohort data from 2002, a period when early and continuous prophylaxis was not yet routine in severe hemophilia, reveal high joint morbidity and the need for surgical intervention even in young adults.^7^

Previous studies have assessed joint health in adults with hemophilia using objective measures such as the Hemophilia Joint Health Score (HJHS) and imaging tools. However, real-world data evaluating joint function, functional independence, and prophylaxis practices across different treatment eras remain limited.^8^

The aim of this study was to evaluate intergenerational differences in joint health among adults with hemophilia and to examine the impact of prophylaxis timing, dose intensity, and treatment adherence on joint outcomes. In particular, the study aimed to explore whether earlier initiation of prophylaxis or dose intensity was more strongly associated with long-term joint outcomes. Joint health was assessed using the HJHS and the Functional Independence Score in Hemophilia (FISH). Findings were interpreted within the context of different treatment eras and historical differences in access to prophylaxis using real-world clinical data.

## Material and Methods

### Participants and Study Protocols

A retrospective cohort study was conducted at a dedicated Hemophilia Center, including adult patients diagnosed with hemophilia A or B who were followed between 2019 and 2021. Patients with a history of inhibitors were excluded. Participants were classified into generational cohorts based on birth year. Individuals born between 1946 and 1964 were categorized as Baby Boomers, those born between 1965 and 1980 as Generation X, participants born between 1981 and 1996 as Millennials (Generation Y), and those born in 1997 or later as Generation Z.^9^ The study sample consisted of 91 patients. Joint health, surgery, and bleeding were assessed in all patients. Of these, 71 patients underwent baseline evaluation with the HJHS and the FISH. One year later, follow-up assessments were performed in a subgroup: 30 patients underwent repeat HJHS and FISH evaluations, while an additional 37 patients underwent FISH only. Consequently, follow-up data were available for 30 patients for HJHS and for 67 patients for FISH. The collected data were analyzed to determine correlations between joint health status, functional independence, and bleeding frequency. Patients were also compared across different generations to assess variations in disease progression and treatment outcomes. In thecountry, factor replacement therapy is administered as prophylaxis at a dose of 15-35 IU/kg per infusion, with patients with hemophilia A receiving treatment 3 times per week and those with hemophilia B twice per week. Patients with incomplete medical records or missing assessment data were excluded. The use of the information was approved through the acquisition of informed written consent. Ethical approval for the study was obtained from Ege University Medical Research Ethics Committee , and all procedures adhered to the principles of the Declaration of Helsinki (Approval No: 21-9T/9; Approval Date: 09.09.2021). This study used AI tools (DeepL and ChatGPT(OpenAI) to improve language, edit grammar, and to assist in the preparation of figures of the manuscript. All output generated by these tools has been reviewed by the authors, edited where necessary, and approved. The authors are solely responsible for the study design, data analysis, interpretation, and conclusions of the manuscript.

### Prophylaxis Regimen and Treatment Practice

Although higher prophylaxis doses are generally recommended in international guidelines, patients at thecenter have historically received relatively low weekly doses of factor replacement therapy (4500-6000 IU), which remain low when adjusted for average body weight. In recent years, prophylaxis regimens have been progressively individualized, with dose adjustments based on patients’ body weight and bleeding frequency. Accordingly, this study evaluated joint health outcomes under long-term low-dose prophylaxis conditions and following individualized dose escalation. Objective clinical measures of joint health were used, and the relationship between joint range of motion and functional capacity was examined. During the study period, extended half-life and non-factor therapies were not reimbursed in thecountry; therefore, all patients received standard short half-life factor concentrates. Individuals diagnosed with hemophilia A received prophylaxis on a thrice-weekly basis, whereas those with hemophilia B received prophylaxis twice weekly. On-demand treatment was administered exclusively for bleeding episodes or surgical indications. During the study period, all patients received standard short half-life (SHL) factor concentrates. In the absence of long half-life products and non-factor therapies, which were not available or covered by reimbursement, all prophylaxis regimens were based on SHL FVIII or FIX.

High-dose prophylaxis consists of administering 25-40 IU/kg of FVIII every 2 days or 40-60 IU/kg of FIX twice weekly (>4000 IU/kg per year). Intermediate-dose prophylaxis is defined as 15-25 IU/kg of FVIII 3 times per week or 20-40 IU/kg of FIX twice weekly (1500-4000 IU/kg per year). Low-dose prophylaxis involves administering 10-15 IU/kg of FVIII or FIX 2 to 3 times per week (1000-1500 IU/kg per year) and is adjusted as needed to prevent bleeding.^5^

### Clinical Assessments

Adherence to the prescribed prophylactic regimen was assessed during routine clinical follow-up visits based on patient self-report. Anamnesis was conducted by the clinical team, including questions regarding the number of prophylactic doses administered, any missed or delayed infusions, and the average frequency of weekly administration. Weekly adherence was calculated in reference to the prescribed dosing schedule, with a week classified as adherent only if all scheduled infusions were completed.

Data on bleeding frequency were limited to joint bleeds and were obtained through patient or caregiver self-report during clinical visits. At each follow-up, patients were asked to report the number of joint bleeding episodes experienced since their previous visit. All information was documented by the clinical team in the medical records. The evaluation was restricted to hemarthroses occurring in major joints, including the knees, elbows, and ankles. The Annual Bleeding Rate (ABR) was calculated by dividing the total number of joint bleeding episodes by the number of months in the reporting period and multiplying the result by 12.

Functional independence was evaluated using FISH, which assesses 8 activities: eating, grooming, dressing, chair transfer, squatting, walking, stair climbing, and running. FISH scores range from 8 to 32, with higher scores indicating greater functional independence. Joint health and mobility were assessed using the HJHS, version 2.1. This tool evaluates the elbows, knees, and ankles based on clinical parameters including swelling, muscle atrophy, joint alignment, crepitus, range of motion, instability, pain, muscle strength, and gait. Total HJHS scores range from 0 (best) to 124 (worst), with individual joint scores ranging from 0 to 20 and a cumulative 6-joint score ranging from 0 to 120.^10^

### Statistics

Quantitative data were analyzed using IBM SPSS Statistics version 24.0 (IBM Corp., Armonk, NY, USA). The Kolmogorov–Smirnov test was used to assess the normality of distribution for continuous variables, and skewness and kurtosis values were examined to support distributional assumptions. For variables that followed a normal distribution, parametric tests were applied, while non-parametric methods were used otherwise. Descriptive statistics are presented as means ± SDs or medians (min-max) for continuous variables, and frequencies (n, %) for categorical variables. Depending on distribution characteristics, paired-sample *t*-tests or Wilcoxon signed-rank tests were used to compare baseline and follow-up scores. Between-group comparisons by prophylaxis dose category (≤ 15 IU/kg, ≤ 25 IU/kg vs. > 25 IU/kg) were performed using independent-samples *t*-tests or Mann–Whitney *U-*tests. Spearman’s correlation was applied to explore associations between HJHS, FISH, and clinical variables. One-way analysis of variance (ANOVA) with Tukey’s HSD was applied for generational comparisons when assumptions were met; otherwise, the Kruskal–Wallis test was used. Two-way ANOVA was employed to assess the interaction effect between generation and prophylaxis adherence, with partial eta-squared (*η*^2^) reported as the effect size. In addition, a univariate General Linear Model (GLM) was conducted to examine the influence of generation, prophylaxis dose, and age at prophylaxis initiation on HJHS, with age, weight, and bleeding frequency included as covariates. McNemar’s test was used to evaluate lateral differences in joint bleeding. A *P* < .05 was considered statistically significant.

## Results

The median age of patients (n = 91) was 38 years (21-69), with a median diagnosis age of 2 years (0-30) and median age at prophylaxis initiation of 27 years (1-62). The mean ABR was 11.1 ± 7.0 (0-35), and the median number of joints affected annually was 2 (0-6) (Table 1). The mean number of bleeding episodes before the prophylaxis dose escalation was 20.1 ± 10.7. During the study period, the median prophylaxis dose was 20.83 IU/kg (IQR, 18.07-23.95). The prevalence of bleeding was observed to be highest in the ankles (54.6%), knees (53.8%), and elbows (47.3%). Baseline demographic and treatment-related characteristics did not differ significantly between patients with and without follow-up HJHS assessments, suggesting no major selection bias in the longitudinal analyses (Table 2). Baseline demographic, clinical, and treatment-related characteristics stratified by generation are summarized in Table 3.

In 16 patients, bleeding episodes significantly decreased after prophylaxis dose escalation (*P* = .007). Initial HJHS scores were assessed in 78 patients (mean 35.4 ± 22.1; range 1-95), and FISH scores in 87 patients (mean 25.1 ± 5.1; range 12-32). HJHS scores differed significantly across generations (*P* < .001), with mean values of 59.2 ± 25.6 in Baby Boomers, 43.7 ± 17.7 in Generation X, 24.9 ± 16.8 in Generation Y, and 13.7 ± 3.2 in Generation Z. The Functional Indepence Score in Hemophilia scores also differed significantly (*P* < .001), ranging from 19.8 ± 5.1 in Baby Boomers to 30.7 ± 1.5 in Generation Z (Table 4).

Subgroup analyses by generation revealed different effects on joint outcomes of prophylaxis dose, treatment adherence, target joint presence, and surgical interventions (Table 5). In the Baby Boomer generation, there was an observed correlation between suboptimal adherence and elevated levels of HJHS and FISH. In Generation X, high-dose prophylaxis and no surgery were associated with lower HJHS, and target joint presence was associated with lower FISH. No significant effect was found in Generation Y. It was not possible to make comparisons with Generation Z due to the limited variability observed (Table 5).

These subgroup and interaction analyses should be interpreted cautiously, as they were exploratory in nature, involved multiple comparisons without formal adjustment for multiple testing, and included small sample sizes in certain generations, particularly Generation Z.

**Figure 1.**
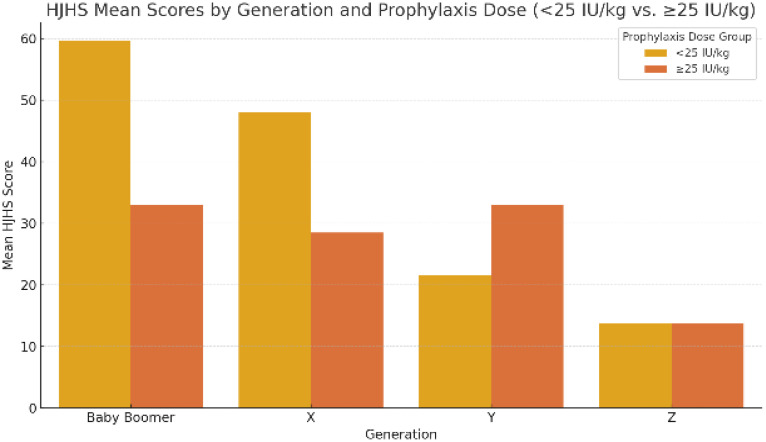
Interaction between generation and prophylaxis dose on Hemophilia Joint Health Scores.

**Figure 2.**
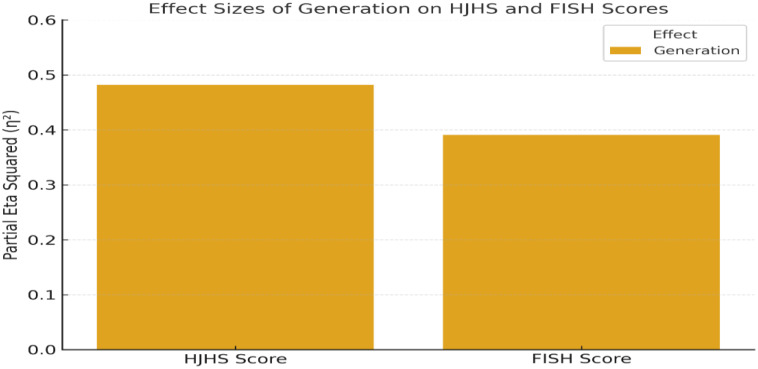
Effect Sizes of Generation on Hemophilia Joint Health Scores and Functional Independence Scores in Hemophilia.

Two-way ANOVA demonstrated significant main effects of generation (*P* < .001, η^2^ = 0.482) and adherence (*P* = .040, η^2^ = 0.100) on HJHS, with a significant interaction (*P* = .013, η^2^ = 0.225). For FISH, the overall model was significant (*P* < .001, adj. R^2^ = 0.375), with generation as a main effect (*P* < .001, η^2^ = 0.391), while adherence and interaction effects were not significant (*P* > .3). A univariate general linear model (GLM) including prophylaxis dose, generation, and age at prophylaxis initiation was significant (*P* < .001, adj. R^2^ = 0.408), with a significant interaction between prophylaxis dose and generation (*P* = .013, η^2^ = 0.123) (Table 5) (Figure 1, Figure 2). Overall, earlier treatment initiation and longer exposure to prophylaxis across treatment eras appeared to be more strongly associated with long-term joint outcomes than prophylaxis dose intensity alone.

**Figure 3.**
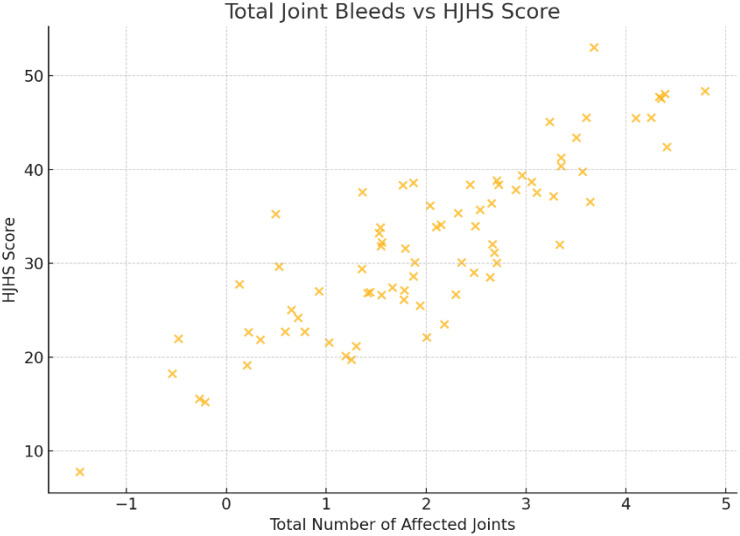
Relationship between total joint bleeds and HJHS score. Scatter plot showing the positive correlation between the number of affected joints and HJHS scores (*r* = .251, *P* = 0.026), indicating worsening joint health with increased joint involvement; HJHS: Hemophilia Joint Health Scores.

**Figure 4.**
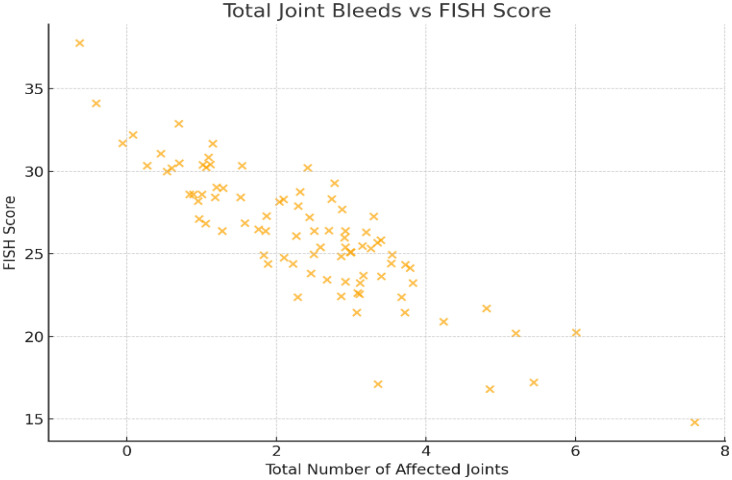
Relationship between total joint bleeds and FISH score. Scatter plot illustrating the negative correlation between the number of affected joints and FISH scores (*r* = −.348, *P* = 0.001), reflecting a decline in functional capacity as joint damage increases; FISH: Functional Independence Score in Hemophilia.

**Figure 5.**
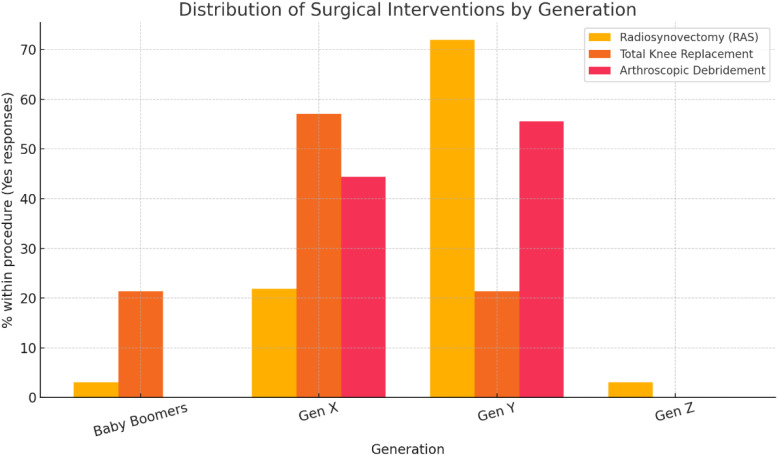
Comparison of surgical interventions across generations.

McNemar’s test revealed no significant side-to-side differences in bleeding frequency for the knees (left: 35.2% vs. right: 35.2%, *P* = 1.000), elbows (left: 30.8% vs. right: 28.6%, *P* = .864), or ankles (left: 39.6% vs. right: 41.8%, *P* = .845) (Table 6). Chi-square analyses showed a significant association between generation and radiosynovectomy (*P* = .013), with 71.9% of cases in Generation Y and 3.1% each in Baby Boomers and Generation Z. For total knee replacement, the overall association was not significant (*P* = .068), but a significant linear trend was observed (*P* = .019), with procedures most common in Generation X (57.1%) and Baby Boomers (21.4%), and absent in Generation Z. No significant association was found for arthroscopic debridement (*P* = .526). Correlation analyses demonstrated a significant positive correlation between the number of joints affected by bleeding and HJHS (*r* = .251, *P* = .026), and a significant negative correlation with FISH (*r* = −0.348, *P* = .001) (Figures 3-4, Figure 5).

Subsequent follow-up assessments demonstrated a mean HJHS of 38.6 ± 19.0 (n = 30) and a mean FISH of 23.2 ± 5.4 (n = 68). A statistically significant baseline difference was observed among the generations (*P* = .038), while changes over time did not reach statistical significance (*P* = .250). A robust correlation was identified between the initial and subsequent HJHS measurements (*r* = .734, *P* < .001). The paired t-test demonstrated a non-significant mean change of −3.63 points (*P* = .188).

## Discussion

The present study demonstrated significant generational differences in joint health and functional outcomes among adults with hemophilia, likely reflecting differences in treatment eras and access to prophylaxis. Younger generations showed better preserved joint integrity and functional independence, whereas Baby Boomer and Generation X cohorts exhibited poorer joint scores and higher rates of surgical intervention. These findings are consistent with previous studies suggesting that delayed initiation and limited access to prophylaxis contribute to increased long-term joint damage.^11^^-^^14^

Despite the intergenerational differences observed at baseline, longitudinal changes in HJHS scores over a 2-year period were not statistically significant. This suggests that short-term follow-up may not adequately capture the trajectory of joint deterioration, which is often gradual and cumulative.^13^ ^,^^15^

The strong positive correlation between baseline and follow-up HJHS scores indicates that initial joint health status remains a strong predictor of long-term outcomes.^11^ ^,^^12^

Although hemophilia A and B are generally considered clinically similar, some studies suggest a milder bleeding phenotype in hemophilia B. However, large cohort studies have shown comparable major bleeding rates between the 2 types. In this study, factor type–specific analyses were not performed due to the limited number of patients with hemophilia B.^16^ ^,^^17^

Later initiation of prophylaxis was associated with worse joint and functional outcomes, reflected by higher HJHS and lower FISH scores. Older cohorts, particularly Baby Boomers and Generation X, demonstrated higher HJHS scores, whereas Generations Y and Z showed better joint preservation, likely reflecting earlier and more sustained prophylaxis exposure.

These findings suggest that earlier and more sustained prophylaxis, rather than dose intensity alone, may be more strongly associated with improved joint outcomes across treatment eras. Adherence was not significantly associated with baseline HJHS or FISH scores, suggesting that historical treatment gaps and access to care may play a greater role in long-term joint outcomes.

Given the limited access to factor concentrates in many regions, low-dose prophylaxis represents a feasible strategy, particularly in resource-limited settings. Evidence from low- and middle-income countries suggests that early and regular low-dose prophylaxis may reduce bleeding frequency and improve clinical outcomes compared with on-demand treatment, although long-term multicenter studies are still needed to clarify optimal prophylaxis strategies.^18^

Importantly, generational comparisons should be interpreted cautiously, as “generation” overlaps with age, historical access to factor concentrates, and timing of prophylaxis initiation. Therefore, the observed differences likely reflect treatment eras rather than intrinsic generational effects, and residual confounding cannot be excluded. Patients aged 20-34 years in earlier cohorts showed greater joint impairment than similarly aged patients in the current Generation Y and Z groups, likely reflecting historical limitations in access to early and sustained prophylaxis.^17^

Due to the limited sample size in the Generation Z group, findings should be interpreted cautiously. Nevertheless, the observed intergenerational differences may reflect historical changes in access to hemophilia treatment and prophylaxis. Older generations experienced more severe joint damage in the context of delayed treatment and limited factor availability, whereas younger generations had earlier and more sustained access to prophylaxis from childhood onward. Consistent with previous literature, these findings suggest that early and sustained prophylaxis may be more strongly associated with long-term joint preservation than dose intensity alone.

Consistent with this interpretation, a 1985 cohort study reported significant joint deterioration among patients aged 21-39 years, corresponding to the Baby Boomer generation at that time. In contrast, thefindings suggest superior joint health among patients within the same age range today, which may be related to earlier initiation, greater regularity, and more individualized dosing of prophylaxis in contemporary practice.^19^

Similarly, a 1999 study reported that major orthopedic surgical procedures were required at relatively young ages (mean age, 38 years). In thecohort, however, surgical interventions appear to have shifted in both timing and type, with major procedures such as total joint replacement being more common in Generation X, while less invasive interventions, including radiosynovectomy, were more frequent in Generation Y. This pattern suggests a change in the timing and magnitude of surgical intervention rather than the complete elimination of surgical need.^18^

In a subset of patients who required an increased prophylaxis dose, a significant reduction in bleeding episodes was observed, indicating that tailored dosing adjustments may improve bleeding control in select cases.

The study also observed significant differences in surgical interventions across generations, including rates of total knee replacement and radiosynovectomy, reflecting the cumulative burden of joint disease in older patients.^11^ ^,^^13^ ^,^^15^

Prior studies similarly show that invasive procedures like joint replacement are more prevalent among older patients, while younger individuals more frequently undergo joint-preserving interventions.^11^ ^,^^20^ ^,^^21^

Additionally, the absence of significant side-to-side differences in joint bleeding (knees, elbows, and ankles) supports a symmetrical distribution pattern regardless of limb dominance.

A study conducted in the Jodhpur region further supports these findings by demonstrating a strong positive correlation between HJHS scores and increasing age, indicating that cumulative joint damage progresses over time.^15^ This is consistent with theresults, where older patients exhibited significantly worse joint health, likely reflecting the cumulative effects of longstanding subclinical or inadequately managed hemarthroses.

In the present study, no significant improvement in joint health or functional outcomes was observed during short-term follow-up, despite improved bleeding control in some patients. Although baseline joint status differed across generations, joint scores remained largely stable over time, suggesting that established joint damage in adults with hemophilia may not regress over short observation periods. Consistent with previous reports, treatment modifications may reduce bleeding frequency, while measurable improvements in joint structure and function likely require longer follow-up, particularly in patients with pre-existing arthropathy.^22^

Finally, international registry data have shown that joint health tends to worsen with age, despite the use of prophylaxis, particularly in weight-bearing joints such as the ankles and elbows.^14^ Thefindings are consistent with this literature, further supporting the notion that hemarthrosis remains a key challenge in long-term hemophilia care.

### Strengths and Limitations

This study’s strengths include a relatively large, single-center hemophilia cohort; the use of objective clinical tools (HJHS, FISH) alongside patient-reported data; and generational comparisons that provide unique insight into the long-term impact of prophylaxis timing, complemented by detailed surgical data. An additional strength is the uniform use of standard short half-life factor concentrates across all patients, which minimizes treatment heterogeneity and reduces confounding related to product type.

Limitations include potential recall bias, the absence of imaging-based assessment for subclinical joint damage, and a relatively short follow-up period that may not fully capture the gradual progression of hemophilic arthropathy. Moreover, not all participants underwent follow-up assessment (HJHS n = 30; FISH n = 67), which may reduce statistical power and introduce selection bias. The single-center design limits generalizability, and residual confounding related to age, historical access to factor concentrates, and timing of prophylaxis initiation cannot be fully excluded. Future studies incorporating longer follow-up periods and imaging methods are needed to ensure a more comprehensive evaluation of joint outcomes. Subgroup and interaction analyses are exploratory in nature; no adjustments were made for multiple comparisons, and the associations should not be interpreted as causal.

## Conclusion

This study suggests that earlier prophylaxis initiation may be associated with better long-term joint outcomes in adults with hemophilia. Delayed prophylaxis initiation was associated with worse joint and functional outcomes, particularly in older generations with historically limited access to treatment. Despite advances in hemophilia care, many adult patients continue to experience the consequences of cumulative joint damage and functional impairment.

These findings should be interpreted cautiously due to the observational design and limited longitudinal follow-up. While early and sustained prophylaxis appeared to be associated with improved joint outcomes, established joint complications in older patients highlight the need for individualized lifelong management. Further studies with larger cohorts, longer follow-up, and emerging therapies are needed to better define optimal treatment strategies.

## Figures and Tables

**Table 1.** Demographic and Clinical Characteristics of Patients with Hemophilia (n = 91)

Characteristic	Hemophilia Type		
Demographics	A (n = 80)	B (n = 11)	All (n = 91)
Age, years, median (min-max)	38 (21-69)	41 (25-64)	38 (21-69)
Age at diagnosis, years, median (min-max)	2 (0-30)	1 (0-6)	2 (0-30)
Age at initiation of prophylaxis, median (min-max)	27 (1-62)	26 (10-54)	27 (1-62)
ABR, median (min-max)	6 (0-35)	5 (3-25)	6 (0-35)
BMI, median (min-max)	26.03 (16.6-40.2)	27.5 (18.6-48.6)	26.17 (16.6-48.6)
Weight median (min-max)	79.5 (50-120)	80 (65-139)	80 (50-139)
Time since prophylaxis initiation, years, median (min-max)	10 (4-26)	10 (8-16)	10 (4-26)
Disease severity			
Severe, n (%)	72 (90.0)	8 (72.7)	80 (87.9)
Moderate, n (%)	5 (6.3)	3 (27.3)	8 (8.8)
Mild, n (%)	3 (3.8)	0 (0.0)	3 (3.3)
Inhibitor status			
Inhibitor-positive, n (%)	0 (0.0)	0 (0.0)	0 (0.0)
Treatment regimen			
Prophylaxis, n (%)	72 (90.0)	11 (100.0)	83 (91.2)
On-demand, n (%)	8 (10.0)	0 (0.0)	8 (8.8)
Prophylaxis doses*, median (IQR**)	20.0 (18.07-23.60)	22.99 (19.50-26.49)	20.83 (18.07-23.95)
Surgical interventions			
Arthroscopic debridement, n (%)	8 (10.0)	1 (9.1)	9 (9.9)
TJR, n (%)	12 (15.0)	2 (18.2)	14 (15.4)
RAS, n (%)	28 (35.0)	4 (36.4)	32 (35.2)
Target joint presence			
Yes, n (%)	73 (91.3)	11 (100.0)	84 (92.3)
No, n (%)	7 (8.8)	0 (0.0)	7 (7.7)

* Prophylaxis dose expressed as IU/kg per infusion.

ABR, annual bleeding rate; BMI, body mass index; ** IQR, interquartile range; TJR, total joint replacement; RAS, radiosynovectomy.

**Table 2.** Baseline Characteristics of Patients with and without Follow-up HJHS Assessments

Variable	Follow-up available (n = 30)	No follow-up (n = 48)	*P*
Generation			.39
Baby Boomer	5 (16.7)	5 (10.4)	
Generation X	7 (23.3)	20 (41.7)	
Generation Y	17 (56.7)	21 (43.8)	
Generation Z	1 (3.3)	2 (4.2)	
Prophylaxis status			.15
Yes	30 (100.0)	43 (89.6)	
No	0 (0.0)	5 (10.4)	
Dose escalation			.12
Yes	8 (26.7)	5 (10.4)	
No	22 (73.3)	43 (89.6)	
Adherence level			.42
Good	20 (66.7)	28 (65.1)	
Moderate	6 (20.0)	5 (11.6)	
Poor	4 (13.3)	10 (23.3)	

Values are presented as n (%). Fisher’s exact test was used when expected cell counts were < 5. No statistically significant differences were observed between groups, indicating a low likelihood of selection or attrition bias.

**Table 3.** Baseline Demographic, Clinical, and Treatment-Related Characteristics of the Study Population Stratified by Generation

Variable	Baby Boomers (n = 12)	Generation X (n = 30)	Generation Y (n = 45)	Generation Z* (n = 4)	P**
Age, years, median (min-max)	61.5 (57-69)	47.0 (41-56)	32.0 (25-39)	23.0 (21-24)	< .001
Age at diagnosis, years, median (min-max)	6.0 (0-30)	2.0 (0-27)	1.0 (0-14)	1.0 (0-3)	.121
Age at initiation of prophylaxis, years, median (min-max)	53.0 (46-62)	36.0 (25-45)	23.5 (5-31)	8.5 (1-16)	< .001
Height (m), median (min-max)	1.65 (1.58-1.82)	1.73 (1.60-1.85)	1.78 (1.65-1.90)	1.71 (1.60-1.80)	< .001
BMI, median (min-max)	28.08 (19.32-40.23)	26.06 (16.98-48.67)	26.12 (16.60-37.04)	22.91 (20.31-25.65)	.118
ABR, median (min-max)	7.5 (0-25)	5.0 (0-25)	7.0 (0-35)	4.5 (2-8)	.454
Hemophilia type					.458
Hemophilia A, n (%)	9 (75.0)	27 (90.0)	40 (88.9)	4 (100)	
Hemophilia B, n (%)	3 (25.0)	3 (10.0)	5 (11.1)	0 (0.0)	
Disease severity					.556
Severe, n (%)	10 (83.3)	27 (90.0)	40 (88.9)	3 (75.0)	
Moderate, n (%)	2 (16.7)	1 (3.3)	4 (8.9)	1 (25.0)	
Mild, n (%)	0 (0.0)	2 (6.7)	1 (2.2)	0 (0.0)	
Treatment regimen					.370
Prophylaxis, n (%)	10 (83.3)	26 (86.7)	43 (95.6)	4 (100.0)	
On-demand, n (%)	2 (16.7)	4 (13.3)	2 (4.4)	0 (0.0)	
RAS					.013
Yes, n (%)	1 (8.3)	7 (23.3)	23 (51.1)	1 (25.0)	
No, n (%)	11 (91.7)	23 (76.7)	22 (48.9)	3 (75.0)	
TJR					.068
Yes, n (%)	3 (25.0)	8 (26.7)	3 (6.7)	0 (0.0)	
No, n (%)	9 (75.0)	22 (73.3)	42 (93.3)	4 (100.0)	
Arthroscopic debridement					.526
Yes, n (%)	0 (0.0)	4 (13.3)	5 (11.1)	0 (0.0)	
No, n (%)	12 (100.0)	26 (86.7)	40 (88.9)	4 (100.0)	

Values are presented as median (min-max) or n (%).

BMI, body mass index; ABR, annual bleeding rate; RAS, radiosynovectomy; TJR, total joint replacement.

*Kruskal–Wallis test or Fisher’s exact test was used where appropriate.

**Generation Z included a limited number of participants and findings should be interpreted cautiously.

**Table 4.** Comparison of HJHS and FISH Scores Across Generations

	HJHS (mean ± SD)	FISH (mean ± SD)	*P* (HJHS)	*P* (FISH)
Baby boomer	59.2 ± 25.6	19.8 ± 5.1		
Generation X	43.7 ± 17.7	23.1 ± 4.2		
Generation Y	24.9 ± 16.8	27.2 ± 4.1		
Generation Z	13.7 ± 3.2	30.7 ± 1.5		
Total	35.4 ± 22.1	25.0 ± 5.1	< .001	< .001

Values are presented as mean ± SD.

HJHS, Hemophilia Joint Health Score; FISH, Functional Independence Score in Hemophilia.

Generation Z included a limited number of participants and findings should be interpreted cautiously.

**Table 5.** ANOVA Results and Effect Sizes (*η*^2^) for Prophylaxis Dose, Adherence, Target Joint, and Surgical Intervention Across Generations

Generations	Variables	HJHS	FISH	*η*^2^ (HJHS-FISH)	Comment
Baby boomer	Prophylaxis doses (<15 U/kg vs. ≥15 U/kg)	—	*P* = .08	—	No comparison possible.
	Prophylaxis doses (<25 U/kg vs. ≥25 U/kg)	0.147	0.083	0.275-0.329	FISH differences clinically relevant, not significant.
	Treatment adherence (good/moderate/poor	<0.001	0.015	0.921-0.700	Limited data; preserved joint health.
	Target joint (yes/no)	—	0.0014	−0.466	Target joints reduce FISH (large effect).
	Surgical intervention (yes/no)	0.313	0.356	0.127-0.86	Better outcomes trend, not significant (small N).
Generation X	Prophylaxis doses (<15 U/kg vs. ≥15 U/kg)	0.419	0.717	0.029-0.006	No significant effect, very small sizes.
	Prophylaxis doses (<25 U/kg vs. ≥25 U/kg)	0.036	0.779	0.178-0.003	Higher doses linked to better HJHS.
	Treatment adherence (good/moderate/poor)	0.240	0.619	0.122-0.041	Limited effect, small-to-moderate on HJHS.
	Target joint (yes/no)	0.356	0.043	0.34-0.143	No effect on HJHS, FISH worse (moderate).
	Surgical intervention (yes/no)	0.049	0.002	0.147-0.293	Surgery group worse HJHS and FISH.
Generation Y	Prophylaxis doses (<15 U/kg vs. ≥15 U/kg)	0.169	0.461	0.055-0.014	HJHS better in low dose, not significant.
	Prophylaxis doses (<25 U/kg vs. ≥25 U/kg)	0.142	0.307	0.062-0.027	No significant dose effect.
	Treatment adherence (good/moderate/poor)	0.832	0.979	0.011-0.001	No effect of adherence.
	Target joint (yes/no)	0.152	0.245	0.056-0.033	No difference; trend toward worse joints.
	Surgical intervention (yes/no)	0.692	0.895	0.004-0.000	No effect; joint health preserved.
Generation Z	Prophylaxis doses (<15 U/kg vs. ≥15 U/kg)			—	No comparisons could be made.
	Prophylaxis doses (<25 U/kg vs. ≥25 U/kg)	—	—	—	Limited data; preserved joint health.
	Treatment adherence (good/moderate/poor)			—	Data insufficient, joints already good.
	Target joint (yes/no)			—	Limited data; preserved joints.
	Surgical intervention (yes/no)	0.766	0.212	0.129-0.893	No difference; joints preserved.

**Table 6.** Distribution of Joint Bleeding by Body Region (n = 91).

Joint	Left side n (%)	Right side n (%)	*P*
Knee	32 (35.2)	32 (35.2)	1.000
Elbow	28 (30.8)	26 (28.6)	.864
Ankle	36 (39.6)	38 (41.8)	.845

Values are presented as n (%). Side-to-side comparisons were performed using the McNemar test. No statistically significant differences were observed between the right and left sides for any joint.

## Data Availability

The data that support the findings of this study are available on request from the corresponding author.
